# Early Screening for Diabetic Retinopathy in Newly Diagnosed Type 2 Diabetes and Its Effectiveness in Terms of Morbidity and Clinical Treatment: A Nationwide Population-Based Cohort

**DOI:** 10.3389/fpubh.2022.771862

**Published:** 2022-04-26

**Authors:** Yu-Chien Chung, Ting Xu, Tao-Hsin Tung, Mingchih Chen, Pei-En Chen

**Affiliations:** ^1^Department of Ophthalmology, Fu Jen Catholic University Hospital, Fu Jen Catholic University, New Taipei City, Taiwan; ^2^Graduate Institute of Business Administration, Fu Jen Catholic University, New Taipei City, Taiwan; ^3^School of Medicine, Fu Jen Catholic University, New Taipei City, Taiwan; ^4^Department Endocrinology and Metabolism, Taizhou Hospital of Zhejiang Province Affiliated to Wenzhou Medical University, Taizhou, China; ^5^Evidence-Based Medicine Center, Taizhou Hospital of Zhejiang Province Affiliated to Wenzhou Medical University, Taizhou, China; ^6^Artificial Intelligence Development Center, Fu Jen Catholic University, New Taipei City, Taiwan; ^7^Institute of Health Policy and Management, National Taiwan University, Taipei, Taiwan; ^8^Taiwan Association of Health Industry Management and Development, Taipei, Taiwan

**Keywords:** newly diagnosed diabetes, type 2 diabetes mellitus, diabetic retinopathy, screen, ophthalmology

## Abstract

**Purpose:**

To characterize the association between the frequency of screening for diabetic retinopathy (DR) and the detection of DR in patients with newly diagnosed type 2 diabetes mellitus (T2DM).

**Methods:**

This nationwide population-based cohort study used data from the National Health Insurance Research Database to identify adult patients who were newly diagnosed with T2DM between 2000 and 2004. Data from their follow-up Diabetic retinopathy (DR) treatments over the next 10 years following diagnosis were also analyzed.

**Results:**

The 41,522 subjects were respectively assigned to a periodic screening group (*n* = 3850) and nonperiodic screening group (*n* = 37,672). Significant differences were observed between the two groups in terms of age, Charlson Comorbidity Index (CCI), sex, DR treatment, and the prevalence of DR. The association between periodic screening and DR treatment, only the elderly, female, and patient with severe CCI status showed the significance in the further stratified analysis.

**Conclusion:**

Periodic screening (annual or biannual screening in the first 5 years) was more effective than nonperiodic screening in detecting instances of DR in the middle-to-advanced aged group but not among younger patients. Screening pattern did not have a significant effect on the likelihood of DR-related treatment during the 5-year follow-up. It appears that a tight screening schedule for the first 5 years after diagnosis with diabetes is not necessary.

## Introduction

Diabetes mellitus (DM) is a growing public health problem. The International Diabetes Federation Diabetes Atlas reported that 463 million adults (9.3%) were living with diabetes in 2019, with estimates of 578 million (10.2%) by 2030 and 700 million (10.9%) by 2045 (1). The global economic burden of diabetes in 2015 was US$1.31 trillion or 1.8% of global gross domestic product (GDP), which was higher in middle-income countries (2). Diabetic retinopathy (DR) is the leading cause of visual impairment among working-age adults; however, increasing awareness and the early identification of DM has ameliorated the problem somewhat (3).

Most standard protocols for the screening of DR recommend annual or biannual dilated retinal examinations for all patients with diabetes (4). Nonetheless, several recent studies on screening and modeling have reported that increasing the screening interval may be more cost-effective for certain groups of patients (5–10).

This retrospective study used data from the Taiwan National Health Insurance Research Database (NHIRD) to assemble five 10-year cohorts by which to analyze the relationship between DR screening frequency and the corresponding incidence of treatment for DR.

## Methodology

### Data Sources

The National Health Insurance Research Database (NHIRD) contains anonymized linked data (e.g., demographic data and medical utilization records), which are available for clinical and epidemiologic research. The coding of diagnostic data is based on the International Classification of Diseases 9th revision Clinical Modification (ICD-9-CM). Note that this retrospective cohort study focused on the “NHIRD-2005 Million Beneficiary” database as the main source of data.

### Research Enrollees

**Figure 1:**
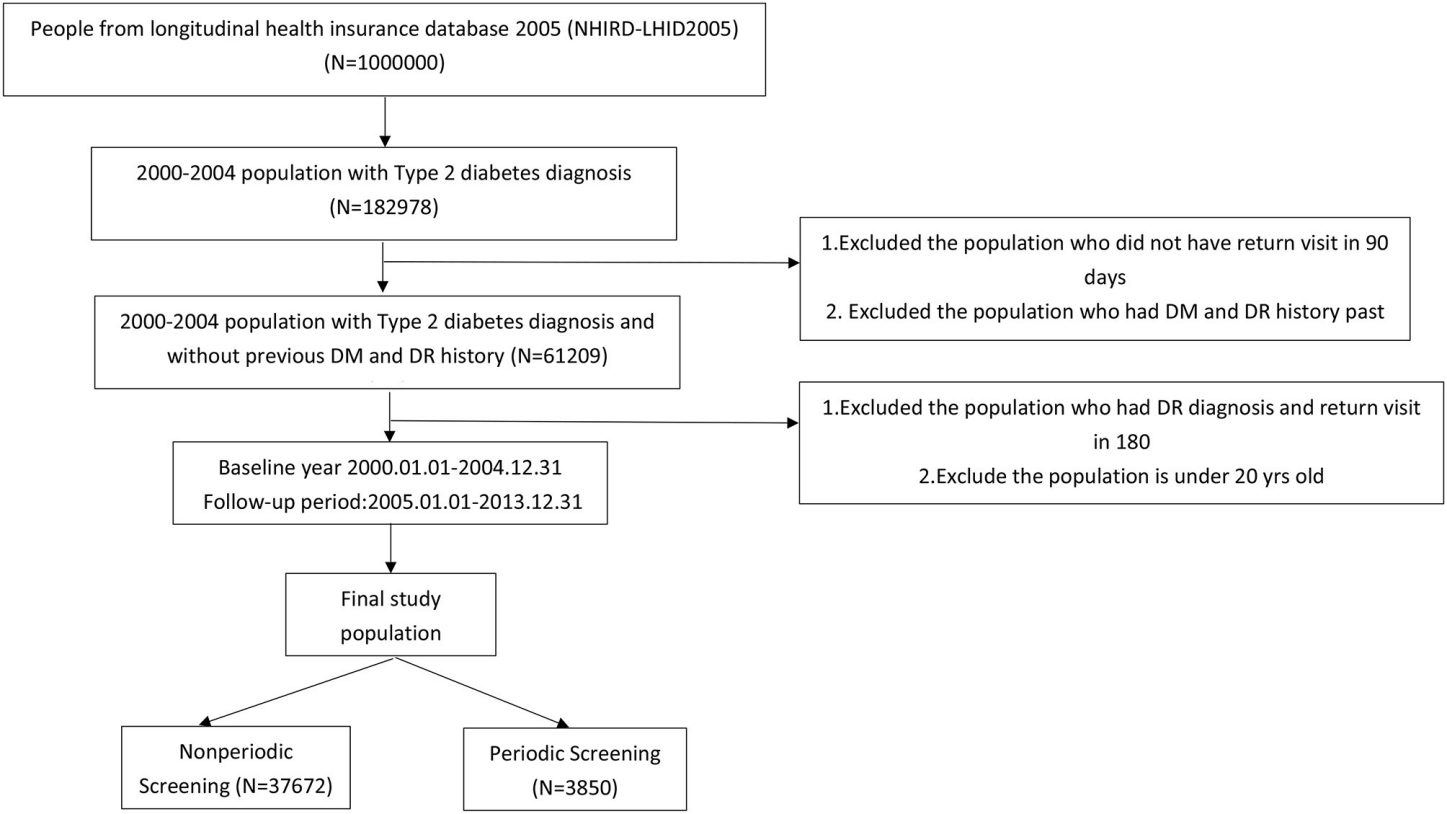
Flowchart.

Enrollees included patients diagnosed for the 1st time with type 2 diabetes mellitus (DM) with at least one subsequent visit within 90 days. The study period was from January 1, 2000, to December 31, 2004. Exclusion criteria included individuals with a history of DM and/or DR in the previous 2 years (ICD-9-CM code 250.xx excluded 250.x1, 250.x3) and those with a DR diagnosis and a return visit within 180 days. The status of enrollees was tracked for 5 years (January 1, 2005, to December 31, 2013; Figure 1). The final study population was then divided into groups according to screening interval, as follows: periodic screening (*N* = 3850) and nonperiodic screening (*N* = 37,672).

### Research Variables

The independent variable in this study was whether subjects underwent periodic screenings (procedure code: 23,501, 23,502, 23,702). The dependent variable was whether subjects diagnosed with DR in the following 5 years (ICD-9-CM code 362.0x) received treatment for DR in the form of laser photocoagulation (procedure code: 60001C−60004C), intravitreal injection (procedure code: 86201C), or/and vitrectomy (procedure code: 86206B−86208B, 86208C, 86212B, 86407B, 86408A, 86408B). Control variables included gender, age, and the Charlson Comorbidity Index (CCI).

### Statistical Analysis

Continuous variables were according to the mean standard deviation, and independent samples *t*-tests were used to examine the mean difference between the two groups. Numbers and percentages were used to describe categorical variables, and simultaneously chi-square tests were performed to assess differences in proportion between the two groups. The Kaplan–Meier method was used to plot survival curves in order to analyze the probability distribution of DR in the two groups. The log-rank test was used to determine whether there was a difference between periodic screening and nonperiodic screening groups. Cox regression analysis was used to examine variables related to DR incidence and treatment with the significant level (α) set at 0.05 (two-sided). The sample size calculations were done, and the sample size needed is 384. SAS 9.4 was used for all statistical analysis.

## Results

### Sample Characteristics

Table 1 lists the demographic characteristics of the enrollees. The mean age of enrollees was as follows: overall (58.0 ± 713.42), periodic screening (60.4 ± 312.07), and nonperiodic screening (57.8 ± 313.53; *P* < 0.001). The baseline CCI scores were as follows: overall (3.5 ± 92.1), periodic screening group (3.9 ± 72.12), and nonperiodic screening group (3.5 ± 52.10; *P* < 0.001). The gender distribution was as follows: overall (20,739; 49.9% males and 20,783; 50.1% females), periodic screening group (1,831; 47.6% males and 2,019; 52.4% females), and nonperiodic screening group (18,908; 50.2% males and 18,764; 49.8% females; *P* = 0.002). The number of patients diagnosed with DR was as follows: overall, (282; 6.8%), periodic screening group (409; 10.6%), and nonperiodic screening group (2,420; 6.4%; *P* < 0.001). The number of patients that received treatment for DR was as follows: overall (1,691; 4.1%), periodic screening group (186; 4.8%), and nonperiodic screening group (1,505; 4.0%; *P* = 0.01).

**Table 1:** Baseline characteristics.

**Variables**	**Total (*N* = 41,522)**	**Periodic screening (*N* = 3850)**	**Nonperiodic screening (*N* = 37,672)**	***P*-value**
	**Mean ±SD or *N* (%)**	**Mean ±SD or *N* (%)**	**Mean ±SD or *N* (%)**	
Age	58.0 ± 713.42	60.4 ± 312.07	57.8 ± 313.53	<0.001
CCI	3.5 ± 92.10	3.9 ± 72.12	3.5 ± 52.10	<0.001
**Sex**
Male	20,739 (49.9)	1,831 (47.6)	18,908 (50.2)	0.002
Female	20,783 (50.1)	2,019 (52.4)	18,764 (49.8)	
DR treatment^*^
Yes	1,691 (4.1)	186 (4.8)	1,505 (4.0)	0.01
No	39,831 (95.9)	3,664 (95.2)	36,167 (96.0)	
**DR**
Yes	2829 (6.8)	409 (10.6)	2,420 (6.4)	<0.001
No	38,228 (93.2)	3,441 (89.4)	35,252 (93.6)	

* *Diabetic retinopathy (DR) treatments*.

Table 2 illustrates the correlation between screening interval and DR treatment as a function of age, gender, or baseline CCI. A significant correlation between periodic screening and DR treatment was observed only in the oldest age group (>65 years; *P* < 0.001). Within females, periodic screening showed a higher proportion in the total amount of DR treatments (*P* = 0.048). Baseline CCI was classified as mild, moderate, and severe, and only acquired significance within the periodic screening group (67,4.9%) which accepted DR treatment more than two times in comparison to the other groups.

**Table 2:** Stratified analysis.

**Variables**		**Periodic screening (*N* = 3850)**	**Nonperiodic screening (*N* = 37,672)**	***P*-value**
		***N* (%)**	***N* (%)**	
**AGE**
20–44 years (*N* = 6,647)	DR treatment^*^			0.44
	Yes	15 (3.6)	272 (4.4)	
	No	405 (96.4)	5,955 (95.6)	
46–64 years (*N* = 20,544)	DR treatment^*^			0.40
	Yes	107 (5.8)	994 (5.3)	
	No	1,743 (94.2)	17,700 (94.7)	
Over 65 years (*N* = 14,331)	DR treatment^*^			<0.001
	Yes	64 (4.1)	239 (1.9)	
	No	1,516 (95.9)	12,512 (98.1)	
**SEX**
Female (*N* = 20,783)	DR treatment^*^			0.048
	Yes	101 (5.0)	765 (4.1)	
	No	1,918 (95.0)	17,999 (95.9)	
Male (*N* = 20,739)	DR treatment^*^			0.13
	Yes	85 (4.6)	740 (3.9)	
	No	1,746 (95.4)	18,168 (96.1)	
**Baseline CCI**
Mild (0–2; *N* = 14,312)	DR treatment^*^			0.34
	Yes	47 (4.7)	714 (5.4)	
	No	960 (95.3)	12,591 (94.6)	
Moderate (3–4; *N* = 14,950)	DR treatment^*^			0.14
	Yes	72 (4.9)	553 (4.1)	
	No	1,396 (95.1)	12,929 (95.9)	
Severe (over 5; *N* = 12,260)	DR treatment^*^			<0.001
	Yes	67 (4.9)	238 (2.2)	
	No	1,308 (95.1)	10,647 (97.8)	

* *Diabetic retinopathy (DR) treatments*.

Tables 3, 4 lists correlations between DR treatment and variables related to the incidence of DR. As shown in Table 3, after controlling for gender, age, and CCI, the risk of developing DR clearly differed as a function of screening pattern. Among older patients, the risk of DR was higher among patients who underwent periodic screening (46–64 years: HR: 1.53, 95% CI: 1.35–1.73; over 65 years: HR: 2.24, 95% CI: 1.89–2.65). Male subjects were shown to be at a lower risk of developing DR (46–64 years: HR: 0.88, 95% CI: 0.81–0.96; over 65 years: HR: 0.78, 95% CI: 0.68–0.90). The risk of developing DR was higher in the middle-aged group (46–64 years) than in the reference group (20–44 years), regardless of gender (male: HR: 1.47, 95% CI: 1.27–1.71; female: HR: 1.60, 95% CI: 1.36–1.88).

**Table 3:** The correlations between Diabetic retinopathy (DR) treatments and variables related to the incidence.

	**Variables**	**Hazard ratio**	**95% CI**		***P*-value**
			**Lower**	**Upper**	
**Age**					
20–44 years	Sex (male vs. female)	0.96	0.79	1.16	0.67
	Periodic screening (yes vs. no)	1.38	0.99	1.92	0.06
46–64 years	Sex (male vs. female)	0.88	0.81	0.96	0.004
	Periodic screening (yes vs. no)	1.53	1.35	1.73	<0.001
Over 65 years	Sex (male vs. female)	0.78	0.68	0.90	0.001
	Periodic screening (yes vs. no)	2.24	1.89	2.65	<0.001
**Sex**					
Male	46–64 years (ref. 20–44)	1.47	1.27	1.71	<0.001
	Over 65 years (ref. 20–44)	0.72	0.59	0.88	0.001
	Periodic screening (yes vs. no)	1.68	1.45	1.94	<0.001
Female	46–64 years (ref. 20–44)	1.60	1.36	1.88	<0.001
	Over 65 years (ref. 20–44)	0.93	0.76	1.13	0.44
	Periodic screening (yes vs. no)	1.72	1.52	1.96	<0.001

**Table 4:** Cox regression of diabetic retinopathy (DR) severity (*N* = 3294).

	**Variables**	**Hazard ratio**	**95% CI**		***P*-value**
			**Lower**	**Upper**	
**Age**					
20–44 years	Sex (male vs. female)	1.04	0.94	1.14	0.45
	Periodic screening (yes vs. no)	1.04	0.74	1.46	0.82
46–64 years	Sex (male vs. female)	1.01	0.96	1.05	0.81
	Periodic screening (yes vs. no)	0.97	0.85	1.10	0.61
Over 65 years	Sex (male vs. female)	1.02	0.95	1.10	0.52
	Periodic screening (yes vs. no)	0.93	0.78	1.10	0.39
**Sex**					
Male	46–64 years (ref. 20–44)	1.13	0.98	1.31	0.09
	Over 65 years (ref. 20–44)	0.95	0.78	1.15	0.59
	Periodic screening (yes vs. no)	0.86	0.74	1.00	0.05
Female	46–64 years (ref. 20–44)	1.06	0.90	1.24	0.51
	Over 65 years (ref. 20–44)	0.90	0.75	1.09	0.29
	Periodic screening (yes vs. no)	1.03	0.91	1.18	0.61

Overall, the risk of DR was higher among patients in the periodic screening group than among those in the nonperiodic screening group (male: HR: 1.68, 95% CI: 1.45–1.94; female: HR: 1.72, 95% CI: 1.52–1.96).

**Figure 2:**
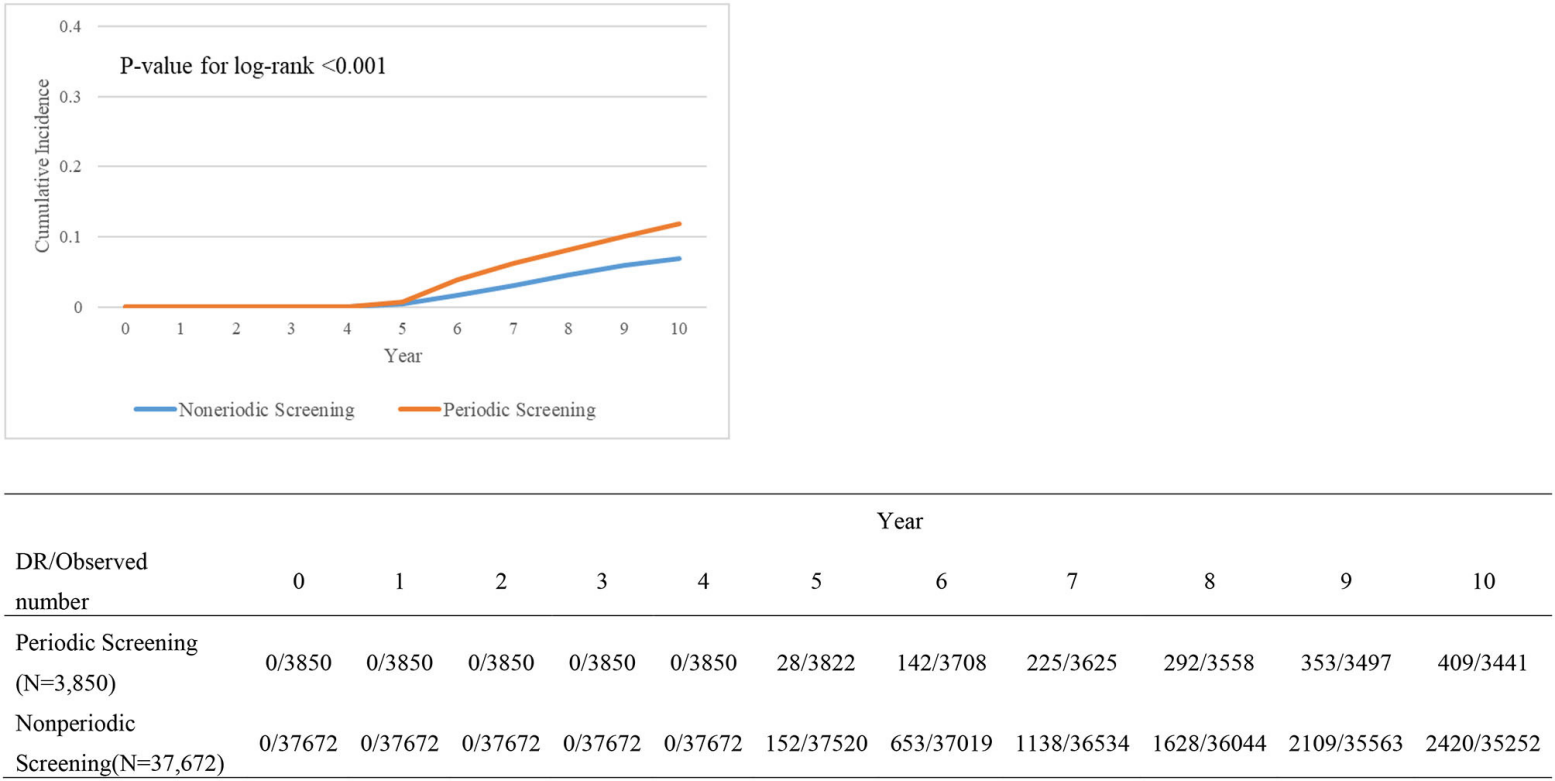
Kaplan–Meier survival plot comparing periodic screening and nonperiodic screening groups.

Table 4 presents the relationship between variables and DR treatments. DM patients with or without periodic screening also differed significantly. However, there was no visible significance when it refers to the included variables. Kaplan–Meier survival plot (Figure 2) shows the cumulative incidence differences between periodic screening and nonperiodic screening groups (*P* < 0.001).

## Discussion

Every year, roughly 1.5 million adults are newly diagnosed with diabetes. In 2018, the age-adjusted incidence was 6.7 per 1000 adults, which is close to the values reported in 2000 (11). Diabetic retinopathy (DR) is characterized by an inflammatory component especially in the early phases (12, 13). With the natural course of DR, patients suffer from nonproliferative stages to proliferative stages that led to extraretinal neovascularization, causing extensive hemorrhage and tractional retinal detachment, and subsequent visual loss. Given the tremendous number of new cases of diabetes yearly and the limited capacity of ophthalmology services, it is important to optimize the screening parameters for DR to prevent sight-threatening DR while balancing the need to control costs. This population-based study sought to characterize the effects of periodic fundus screening for DR among patients newly diagnosed with type 2 DM in terms of DR incidence and DR-related treatment over the first 5 years after diagnosis. We examined 41,522 patients from five longitudinal cohorts.

In the current study, only 9.2% of newly diagnosed diabetic patients underwent periodic fundus screening in the first 5 years after diagnosis. More than half of these patients (59%) were not subjected to ophthalmic screening, as reported in previous studies (14, 15). The overall incidence of DR in the 2nd 5-year period was 7.9%. Note that the incidence of DR in this study was close to that recorded in Chinese populations (16, 17) and other ethnic groups (18). Patients that underwent periodic screening presented a higher incidence of DR in the 2nd 5-year period. The Cox regression results in Table 3 revealed that periodic screening was the main variable contributing to the detection of DR in middle-to-advanced aged patients. Screening patterns did not have a significant effect among younger patients. Note that screening is meant to identify previously unknown cases of DR. Thus, it is reasonable to expect that periodic screening would result in a higher incidence of DR. Our data further narrow the group benefit from periodic screening to middle-to-advanced aged patients. Previous studies have identified a number of major risk factors for DR, including duration of diabetes, hyperglycemia (19), and hypertension (20). Advanced age has also been implicated in DR (18, 21, 22). In one previous hospital-based study of 127 patients newly diagnosed with diabetes, multivariate analysis revealed age and HbA1c levels as the only factors associated with DR incidence, wherein the likelihood of developing DR increased by 11% per year in the age of the patients (18).

Treatment for DR is required in 4.1% of patients newly diagnosed with diabetes (i.e., advanced DR). In the current study, the proportion of cases requiring treatment was significantly higher in the periodic screening group than in the nonperiodic group (Table 1, periodic: nonperiodic = 4.8%:4.0%, *P* = 0.01). Further analysis revealed that the major risk factors for treatment were advanced age (*P* < 0.001) and female gender (*P* = 0.048). We also examined the relationship between screening and the incidence of treatment in patients diagnosed with DR during the 2nd 5-year period. The Cox regression results in Table 4 revealed that none of the variables (age, gender, and pattern of screening) had a significant effect on the likelihood of receiving treatment for DR. In one previous study, the median time to DR progression was 7.6 years (23). One retrospective cohort study of 4513 patients reported that progression to advanced DR is infrequent within the first 10 years of diabetes (24). The Liverpool Diabetic Eye Study (4770 patients newly diagnosed with diabetes) reported on the annual incidence of sight-threatening DR among patients without retinopathy at baseline: first year (0.3%), fifth year (1.8%), and cumulative incidence at 5 years (3.9%) (10). Recent hospital-based and population-based studies have reported that only 0.3–5% of newly diagnosed diabetes patients required treatment for DR (25–27). The low overall incidence of treatment in our nonperiodic screening group can be attributed to volunteer bias, wherein a large proportion of the patients made no effort to undergo ophthalmic examinations during the study period. Table 5 lists the relevant studies related to DR screening.

**Table 5:** Characteristics of the relevant studies.

**First author, year**	**Region**	**Study subject**	**Newly diagnosed diabetes**	**Follow up period**	**Age (Mean ±SD, years)**	**Sex *N* (%)**	**Results/Conclusion**
Liu et al. (7)	Taiwan	795	No	January 1990–December 1992	59.7 ± 8.32	Female: 441 (55.5); Male: 354 (44.5)	1. An annual screening program, a biennial screening regime and a 4-yearly screening regime can lead to 54% (95% CI: 44–62%), 51% (95% CI: 41–59%), and 46% (95% CI: 36–54%) reductions in blindness, respectively
Younis et al. (10)	United Kingdom	4,770	Yes, but number not specified	6 years	(median [IQR]) NDR: 63.4 (56.1–69.8); Background DR: 64.7 (57.9–71.1); Mild preproliferative DR: 65.0 (57.6–71.8)	Female: 2,116 (44.4); Male: 2,654 (55.6)	1. Yearly incidence of sight-threatening DR in patients without DR at baseline was 0.3% (95% CI 0.1–0.5) in the first year, rising to 1.8% (1.2–2.5) in the fifth year; cumulative incidence at 5 years was 3.9% (2.8–5.0) 2. Rates of progression to sight-threatening DR in year 1 by baseline status were: background 5.0% (3.5–6.5), and mild preproliferative 15% (10.2–19.8) 3. Mean screening intervals by baseline status were: no DR 5.4 years (95% CI 4.7–6.3), background 1.0 years (0.7–1.3), and mild preproliferative 0.3 years (0.2–0.5)
Agarwal et al. (28)	India	301	Yes (*n* = 128)	June 2003–September 2004	Group I (Targeted Screening): 54 ± 11; Group II (Newly Diagnosed): 52 ± 12	Female: 148 (49.2); Male: 153 (50.8)	1. The occurrence of DR was 6.35% (95% CI, 2.5–9.5) in Group I and 11.71% (95% CI, 5.6–16.4) in Group II. (*P* > 0.05), including sight-threatening retinopathy, in rural versus urban population and in Group I versus Group II 2. Group II with systolic blood pressure (BP) >140 were more likely to have retinopathy (*P* = 0.02)
Namperu malsamy et al. (22)	India	25,969	Yes (*n* = 1478)	August 2005–March 2006	N/A	Female: 13,525 (52.1%); Male: 12,444 (47.9%)	1. Among the subjects screened for DM, 2802 (10.8%, 95% CI 9.3–12.2%) were found to have DM 2. DR was detected in 298 (1.2%) of included subjects. The age-gender-adjusted prevalence of DR is 0.05% (95% CI 0.04–0.06%) for rural and 1.03% (95% CI 0.89 to 1.12%) for urban areas 3. The overall age–gender-cluster adjusted prevalence of DR was 0.74% (95% CI 0.66–0.83%). DR was present in 12.2% (95% CI 10.4 to 14.1%) of the DM population
Agardh et al. (9)	Sweden	1,322	Not specified	3 years	55 ± 12	N/A	1.73% were still without retinopathy after 3 years, and 28% had developed mild or moderate retinopathy, but none developed severe nonproliferative or proliferative retinopathy
Lee et al. (29)	China	3,510	Yes (*n* = 3510)	2006–2009	59.5	Female: 1,811 (51.6); Male: 1,699 (48.4)	1. The prevalence of DR was 18.2% (639 patients) among the recently diagnosed DM patients 2. In 639 patients with DR, 7% were with significant macular edema
Wang et al. (30)	China	368	Yes (*n* = 247)	2006–2007	N/A	Female: 233 (63.3); Male: 135 (36.7)	1. The age-standardized prevalence of DR was 43.1%. In multivariable-adjusted logistic regression models for all DM participants, independent risk factors for DR were longer duration of diabetes (OR = 3.07, 95% CI 1.94–4.85), higher FPG levels (OR 1.17; 95% CI: 1.08–1.27) and higher systolic BP (OR 1.22; 95% CI: 1.08–1.37) 2. For newly diagnosed diabetes, the only significant factor of DR was higher FPG levels (OR 1.17; 95% CI 1.05–1.29, per mmol/l increase)
Looker et al. (27)	United Kingdom	51,526	Yes (*n* = 51,526)	Jan. 2005–May 2008	61.8 ± 12.8	Female: 22,950 (45); Male: 28,576 (55)	1. The prevalence at first screening of any retinopathy was 19.3%, and for referable retinopathy it was 1.9%. For individuals screened after a year the prevalence of any retinopathy was 20.5% and referable retinopathy was 2.3%
							2. Any retinopathy at screening was associated with male sex (OR 1.19, 95% CI 1.14–1.25), HbA1c (OR 1.07, 95% CI 1.06–1.08), systolic BP (OR 1.06, 95% CI 1.05, 1.08), time to screening (OR for screening >1 year post diagnosis = 1.12, 95% CI 1.07–1.17) and obesity (OR 0.87, 95% CI 0.82–0.93)
Hayat et al. (26)	Pakistan	100	Yes (*n* = 100)	Nov 2009–Jun 2010	45.1 ± 3.2	Female: 60 (60.0); Male: 40 (40.0)	1.17% of type 2 DM patients had retinopathy within 1 month of diagnosis 2. Background retinopathy was predominant (12%) followed by pre-proliferative (4%) and proliferative (1%) lesions
Jammal et al. (18)	Jordan	127	Yes (*n* = 127)	6 months	49.7 ± 10.0	Female: 46 (36.2); Male: 81 (63.8)	1.7.9% DR in the included subjects 2. Patients with DR, 40% with significant macular edema 3. The odds of DR increased by 11% for each 1 year increase in age (odds ratio [OR] 1.11; 95% confidence interval [CI] 1.02–1.20). For each 1% increase in HbAlc, the odds of DR increased by 43% (OR 1.43; 95% CI 1.09–1.88)
Xu et al. (17)	China	2602	Not specified	10 years	64.6 ± 9.7	N/A	1.109 subjects (39 men) developed new DR with an incidence of 4.2% (95% CI: 3.45–5.03) 2. In multiple logistic regression analysis, incident DR was associated with higher HbA1c value (*P* = 0.001; OR = 1.73 95% CI: 1.35–2.21), longer duration of DM (*P* = 0.001; OR: 1.16; 95% CI: 1.10,1.22), higher serum concentration of creatinine (*P* = 0.02; OR: 1.01; 95% CI: 1.002,1.022), lower educational level (*P* = 0.049; OR: 0.74; 95% CI: 0.55,0.99), higher estimated cerebrospinal fluid pressure (*P* = 0.038; OR: 1.10; 95% CI: 1.01,1.22), and shorter axial length (*P*, 0.001; OR: 0.48; 95% CI: 0.33, 0.71)
Ponto et al. (25)	Germany	285	Yes (*n* = 285)	N/A	N/A	Female: 114 (40.0); Male: 171 (60.0)	1. The weighted prevalence of DR in screening-detected type 2 DM was 13.0%; 12% of participants had a mild non-proliferative DR and 0.6% had a moderate nonproliferative DR 2. DR was proliferative in 0.3%. No cases of severe non-proliferative DR or diabetic maculopathy were found
Tóth et al. (31)	Germany	3,523	Yes (*n* = 44)	April–July 2015	N/A	Female: 2,250 (63.9); Male: 1,273 (36.1)	1.20% of participants with known DM had a blood glucose level ≥200 mg/dL, and 27.4% had never had an ophthalmological examination for DR 2. Prevalence of DR and/or maculopathy was 20.7% and prevalence of sight-threatening DR was 4.3% in one or both eyes among participants with DM
Al-Zamil et al. (32)	Saudi Arabia	112	Yes (*n* = 112)	Jan. 2012–Jan. 2015	51.2 ± 5.3 (DR: 53.4 ± 6.4)	Female: 62 (55.4); Male: 50 (44.6)	1. DR was in seven patients (6.25%) 2. Two patients (28.6%) presented with bilateral clinically significant macular edema requiring further treatments 3. At the time of type 2 DM diagnosis, uncontrolled HbA1C levels were significantly associated with the presence of retinopathy (*P* = 0.045)
Chatziralli et al. (33)	United Kingdom	1,062	Yes (*n* = 1,062)	2 years	56.0 ± 10.9	Female: 477 (44.9); Male: 585 (55.1)	Risk factors that remained significantly associated with DR presence at the multivariate analysis were male sex, any cardiovascular event, HbA1c, and IL-1RA
Rudnisky et al. (23)	Canada	980	Not specified	10 years	DR status Progressed: 54.9 ± 12.7; Stable: 53.6 ± 13.7	Female: 829 (84.6); Male: 151 (15.4)	1. At baseline, most patients had no DR (*n* = 777, 79.3%) whereas 203 people (20.7%) had either nonproliferative DR (*n* = 179, 18.3%) or proliferative DR (*n* = 24, 2.5%) 2. Two-step progression occurred in 163 patients (16.6%), with only a minority of these individuals progressing to proliferative DR (*n* = 23). The median time to progression was 7.6 years. Multivariate Cox regression demonstrated that elevated hemoglobin A1C (hazard ratio [HR] = 1.42; *P* < 0.0001) and systolic BP (HR = 1.24; *P* = 0.009) were independent predictors of progression of DR
Voigt et al. (24)	Germany	2,272	Not specified	1987–2014	65.4 ± 12.6	N/A	1.25.8 % of the patients had DR (20.2 % nonproliferative, 4.7 % proliferative, 0.7 % were not classified, 0.1 % blindness) 2. The prevalence of DR in dependence on diabetes duration was 1.1 % at diagnosis, 6.6 % after 0 < 5 years, 12 % after 5 < 10 years, 24 % after 10 < 15 years, 39.9 % after 15 < 20 years, 52.7 % after 20 < 25 years, 58.7 % after 25 <30 years and 63 % after ≥ 30 years 3. In a subset of 586 (25.7 %) patients with retinal photography of 3 consecutive years 7.0 % showed deterioration after 1 and 12.2 % after 2 years; 2.6 % improved after 1 and 2.8 % after 2 years. 201 (34.3 %) of this group had < 10 years diabetes and lower deterioration (4.5 % worsened after one and 9.5 % after 2 years). Their retinopathy mainly transformed from no retinopathy to nonproliferative. Four patients (2.0 %) developed proliferative retinopathy
Cui et al. (34)	China	1,500	Yes (*n* = 936)	September 2011–February 2012	59.5 ± 11.1	Female: 886 (59.1); Male: 614 (40.9)	1. Standardized prevalence rate of DR was 18.2% for all patients with diabetes, 32.8% for the patients with previously diagnosed diabetes, and 12.6% for newly diagnosed patients with T2DM. The prevalence rate of male DR was significantly higher than that of female DR (23.0% vs 14.1%, *P* < 0.001) 2. The prevalence rates of vision-threatening DR, diabetic macular oedema, and clinically significant macular oedema were 2.5%, 2.8% and 0.9%, respectively. Male, higher education level, longer duration of DM, higher systolic BP and glycosylated hemoglobin were independent risk factors for DR development
Hao et al. (35)	China	947	Yes (*n* = 947)	December 2018–April 2019	No DR: 53.3 ± 11.7; DR: 52.9 ± 11.1	Female: 381 (40.2); Male: 566 (59.8)	1. BMI was shown to be a related factor for DR in patients with newly diagnosed diabetes (OR = 0.592, *P* = 0.004). When BMI was ≥28 kg/m2, heavy smoking was associated with DR (OR = 2.219, *P* = 0.049) 2. There was a negative correlation between DR and the age of diagnosis of diabetes ≥60 years (OR = 0.289, *P* = 0.009)
Hwang et al. (36)	Korea	380	Yes (*n* = 380)	Jan. 2013–Jan. 2018	No DR: 51.61 ± 12.48; DR: 50.78 ± 10.21	Female: 159 (41.8); Male: 221 (58.2)	1.40 (10.53%) patients had DR at the initial ophthalmologic examination
							2. Glycated hemoglobin, fasting plasma glucose, urine albumin to creatinine ratio, and urine microalbumin level were significantly higher in DR patients than in patients without DR 3. In the multivariate logistic regression analysis, high HbA1C was a significant risk factor for the presence of DR at new T2DM diagnosis (OR: 2.372; *P* < 0.001). HbA1C, FPG, UACR, and urine microalbumin levels showed significantly positive correlations with DR severity
Shah et al. (37)	United Kingdom	11,399	Yes (*n* = 11,399)	2005–2009	Median (IQR); At baseline; No DR: 60 (51–69); DR: 61 (52–69)	Female: 5,116 (45) Male: 6,283 (55)	1. Baseline retinopathy prevalence was 18% (*n* = 2048) versus 37% in UKPDS. At 7 years, 11.6% (*n* = 237) of those with baseline retinopathy had progression of retinopathy 2. In those without baseline retinopathy, 46.4% (*n* = 4337/9351) developed retinopathy by 7 years. Retinopathy development (OR: 1.05; 95%CI: 1.02–1.07) and progression (OR: 1.05 [1.04–1.06]) at 7 years was associated with higher HbA1c at diabetes diagnosis. Obesity (OR: 0.88 [0.79–0.98]) and high socioeconomic status (OR: 0.63; 0.53–0.74) were negatively associated with retinopathy development at 7 years

The strength of this study lies in the evidence derived from a large population-based database. Note that DR treatment was selected as the main outcome measurement due to the fact that the coding for treatment in the NHIRD system is more precise than that for diagnosis. Furthermore, DR treatment reflects the actual economic burden on the healthcare system. Nonetheless, this study has several limitations. We did not examine the related parameters, such as glycemic index, insulin use, lipid levels, or blood pressure, hence the level of glucose control and the co-existence of other risk factors cannot be included in the analysis. Second, the follow-up for DR was limited to 5 years, thereby preventing analysis of long-term effects. Third, the limited availability of data imposed a number of difficulties in defining cases of severe DR and subgroup analysis. Fourth, we encountered potential overlaps in data pertaining to age-related macular degeneration and retinal vein occlusion. Fifth, the diagnosis of DM in this study may have been delayed, due to difficulties in defining the specific day of diagnosis using the NHRI database. Further analysis over a longer period will be needed to gain a more comprehensive understanding of the effects of periodic screening.

In summary, periodic screening of patients newly diagnosed with T2DM (annual to biannual screening in first 5 years) was shown to increase the detection of DR among middle-to-advanced aged patients; however, it was not correlated to the incidence of DR-related treatment. Periodic screening had no effect on detection rates among younger patients (<45 years).

We recommend a longer screening interval for younger patients newly diagnosed with diabetes. From the perspective of public health policy, this study suggests that a tight screening schedule probably is not necessary for all patients during the first 5 years after diagnosis, however the ophthalmologist should consider very careful the clinical history of each patient and anticipate the follow-up if necessary.

## Data Availability Statement

The original contributions presented in the study are included in the article/supplementary materials, further inquiries can be directed to the corresponding author.

## Author Contributions

Y-CC: writing manuscript. T-HT: supervise and professional statistical counseling. P-EC: data analysis and drafting manuscript. TX and MC participated and performed data synthesis of the revised stage. All authors contributed to the article and approved the submitted version.

## Conflict of Interest

The authors declare that the research was conducted in the absence of any commercial or financial relationships that could be construed as a potential conflict of interest.

## Publisher's Note

All claims expressed in this article are solely those of the authors and do not necessarily represent those of their affiliated organizations, or those of the publisher, the editors and the reviewers. Any product that may be evaluated in this article, or claim that may be made by its manufacturer, is not guaranteed or endorsed by the publisher.
